# Diagnostic Evolution From Channelopathy to Idiopathic Ventricular Fibrillation After Aborted Sudden Cardiac Death

**DOI:** 10.1016/j.jaccas.2026.107624

**Published:** 2026-03-28

**Authors:** Faizan Butt, Priyank Chokshi, Paul Matthew Badua, Janish Kothari

**Affiliations:** a Department of Cardiology, Rutgers Jersey City Medical Center, Jersey City, New Jersey, USA; b Department of Pharmacology, Rutgers Jersey City Medical Center, Jersey City, New Jersey, USA

**Keywords:** cardioversion, genetics, ventricular fibrillation, ventricular tachycardia

## Abstract

**Background:**

Idiopathic ventricular fibrillation (IVF) is a life-threatening condition with no identifiable structural or metabolic etiology. It is a diagnosis of exclusion and accounts for a significant proportion of unexplained sudden cardiac death (SCD) in young healthy individuals.

**Case Summary:**

In a 23-year-old woman initially experiencing seizure-like activity, long QT syndrome was later diagnosed. Following placement of an implantable cardioverter-defibrillator, subsequent evaluation, including cardiac magnetic resonance, negative 51-gene arrhythmia panel, and subcutaneous cardiac monitor implantable loop recorder, confirmed the spontaneous episode of ventricular fibrillation. In the absence of a persistent long QT syndrome phenotype, the findings were most consistent with IVF.

**Discussion:**

This case underscores the importance of systematic reassessment and high clinical suspicion to establish IVF and guide timely management.

**Take-Home Message:**

IVF is a diagnosis of exclusion requiring comprehensive evaluation and management with implantable defibrillators and antiarrhythmic therapy for preventing recurrent episodes.

## Graphical Abstract

**Figure:**
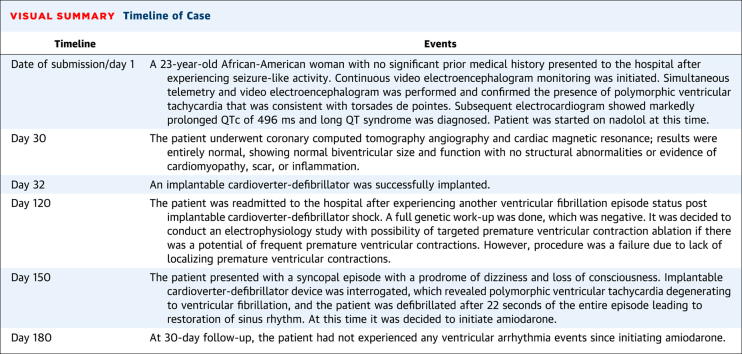

## History of Presentation

A 23-year-old African-American woman with no significant prior medical history presented to the hospital emergency department after experiencing seizure-like activity. She reported no preceding chest pain or palpitations. On prior evaluation for a seizure disorder, magnetic resonance imaging and computed tomography of the brain were normal, and she was prescribed on levetiracetam.

**Take-Home Message**

• IVF is a diagnosis of exclusion requiring comprehensive evaluation and management with ICDs and antiarrhythmic therapy for prevention of recurrent episodes.

Initial work-up in the hospital included a 12-lead electrocardiogram (ECG) that showed normal cardiac rhythm. Continuous video electroencephalogram (EEG) monitoring was also initiated. During her stay, she experienced another episode of seizure-like activity. Simultaneous telemetry and video EEG were performed that confirmed the presence of polymorphic ventricular tachycardia that was consistent with torsades de pointes (TdP), whereas the EEG showed no epileptiform activity. A subsequent ECG showed a markedly prolonged QTc of 496 ms, and long QT syndrome (LQTS) was diagnosed.

## Past Medical History

The patient had no prior medical history of cardiac disease. Family history also was negative for sudden cardiac deaths or arrhythmias; her parents and siblings were healthy. She was not taking any QT-prolonging medications other than recently initiated antiepileptic therapy.

## Differential Diagnosis

The initial presentation with loss of consciousness, documented TdP, and QT prolongation prompted consideration of congenital LQTS as the leading diagnosis. Other differential diagnoses included acquired QT prolongation due to transient or reversible factors, catecholaminergic polymorphic ventricular tachycardia, and Brugada syndrome. Idiopathic ventricular fibrillation (IVF) was subsequently considered after exclusion of structural heart disease, normalization of the QT interval, and negative comprehensive genetic testing.

## Investigations

Initial ECG on admission showed sinus rhythm with a QTc of 393 ms. Following the documented TdP event and repeat ECG demonstrating a prolonged QTc to 496 ms, congenital LQTS was diagnosed. The Schwartz score was calculated to be 5 (3 points for QTc ≥480 ms, 2 points for TdP), indicating a high probability of LQTS.

A comprehensive cardiac evaluation was undertaken to rule out structural causes including transthoracic echocardiogram and cardiac magnetic resonance. The results were entirely normal, showing normal biventricular size and function with no structural abnormalities or evidence of cardiomyopathy, scar, or inflammation.

To further evaluate for an inherited arrhythmia substrate, a comprehensive 51-gene arrhythmia and cardiomyopathy panel was sent. The results were negative for pathogenic variants in all genes tested, including those associated with LQTS, Brugada syndrome, catecholaminergic polymorphic ventricular tachycardia, and others. This negative genetic result prompted a re-evaluation of the LQTS diagnosis.

**Figure 1:**
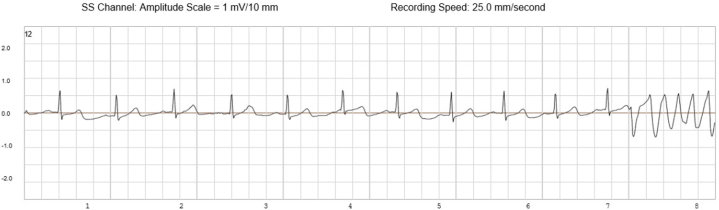
Baseline Sinus Rhythm With Normal QT Interval Recorded by the Implantable Loop Recorder Just Before Ventricular Fibrillation

**Figure 2:**
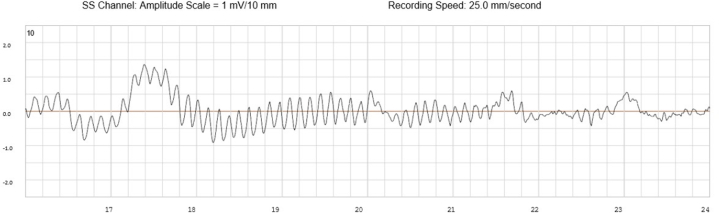
Abrupt Onset of Disorganized, Polymorphic Ventricular Activity Consistent With Primary Ventricular Fibrillation

**Figure 3:**
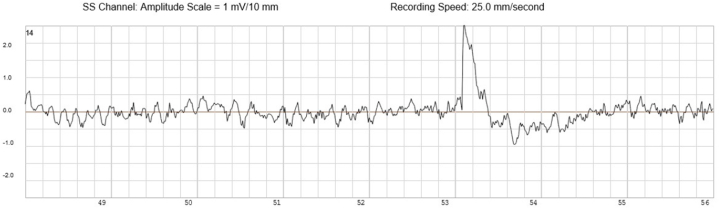
Continuation of Chaotic Rhythm as Sustained Coarse Ventricular Fibrillation

An implantable cardioverter-defibrillator (ICD) was implanted for secondary prevention along with an implantable loop recorder (ILR) for long-term rhythm characterization. The critical diagnostic confirmation came from the routine interrogation of these implanted devices. The ILR data documented a spontaneous, symptomatic arrhythmic event starting with sinus rhythm with a normal QT interval (Figure 1) with an abrupt transition from a single organized rhythm directly into a chaotic, disorganized rhythm (Figures 2 and 3) consistent with primary ventricular fibrillation. This rhythm persisted until termination by a successful ICD shock or defibrillation.

## Management

Following the diagnosis of TdP with QT prolongation, all antiepileptic drugs were discontinued. In the absence of any structural heart disease on advanced imaging along with the negative comprehensive genetic test and the lack of a persistently prolonged QT interval, the findings were more consistent with IVF in the patient. The management of IVF presents a unique challenge as it is a diagnosis of exclusion, and in contrast to LQTS or catecholaminergic polymorphic ventricular tachycardia, there are no genotype-specific therapy guidelines. Therefore our approach was pragmatic and based on the principles of secondary prevention.

### ICD Therapy

The patient received an ICD, which is the nonnegotiable cornerstone of therapy, a decision unequivocally validated by the delivery of a life-saving shock.

### Pharmacologic Therapy

Pharmacologic therapy was initiated to suppress recurrent ventricular arrhythmias and prevent ICD shocks. The choice of amiodarone in conjunction with nadolol represents an aggressive, multimechanism approach for secondary prevention. Nadolol provides beta blockade, which may suppress adrenergically mediated triggers, whereas amiodarone, with its complex class III effect, increases the refractory period across the myocardium. The patient was started on a daily regimen of amiodarone to provide a multichannel antiarrhythmic effect and reduce the potential for recurrent ventricular fibrillation episodes along with ICD. This combination is often reserved for patients at highest risk of recurrent events or electrical storms.

## Outcome and Follow-Up

At follow-up, the patient remained asymptomatic, with no further episodes of syncope. Device interrogation demonstrated no recurrent sustained ventricular fibrillation events requiring ICD therapy. She continues on close outpatient follow-up with electrophysiology, with ongoing ICD surveillance and medical therapy.

## Discussion

This case not only describes a rare diagnosis, but also provides a critical narrative of the evolving diagnostic pathway for survivors of sudden cardiac death in the modern era. This case report illustrates how the integration of prolonged rhythm monitoring and comprehensive genetic testing can refine a diagnosis from a presumed specific channelopathy toward a working diagnosis of IVF^1^ within the limits of current diagnostic capabilities.^2^^,^^3^ Our discussion focuses on 3 pivotal aspects: the diagnostic odyssey from phenotype to genotype, the critical role of continuous monitoring, and the subsequent implications for clinical management.

### Diagnostic Odyssey: From Phenotypic Mimicry to Genotypic Exclusion

The initial presentation of this patient, featuring TdP and QT prolongation, represents a robust and clinically meaningful phenotype strongly suggestive of a repolarization disorder such as LQTS. This phenotype warranted serious consideration and comprehensive evaluation. However, phenotypic expression in the acute setting may be influenced by transient or acquired factors, leading to arrhythmic mimicry, in which a nonspecific electrical instability manifests with the hallmark signature of a known disease. Several transient factors could have contributed to this initial phenotype, including the proarrhythmic effects of previously administered antiepileptic drugs or the electrophysiological turmoil following a cardiac arrest.

The definitive pivot in the diagnostic journey came from the comprehensive 51-gene arrhythmia panel. A negative genetic test for a clinically robust LQTS case occurs in 20% to 25% of patients (often termed genotype-negative LQTS).^4^^,^^5^ However, in this instance, the negative result, combined with the subsequent normalization of the QT interval in the absence of provoking factors, allowed for a diagnostic re-evaluation. It effectively decoupled the final diagnosis from LQTS.^6^ Importantly, a negative gene panel does not exclude the presence of polygenic contributions, regulatory variants, or pathogenic mutations yet to be discovered, underscoring the inherent limitations of contemporary genetic testing. As outlined in Table 1, the systematic exclusion of all major channelopathies through genetic testing allowed the diagnosis^7^ to evolve from presumptive LQTS to a diagnosis of IVF by informed exclusion rather than absolute certainty.

**Table 1:** Systematic Exclusion of Channelopathies via Genetic Testing

Syndrome Excluded	Key Genes Analyzed	Phenotype Overlap	Results
Long QT syndrome	*KCNQ1, KCNH2, SCN5A, ANK2, KCNE1*	Torsades de pointes, prolonged QTc	Negative
Brugada syndrome	*SCN5A, CACNA1C, CACNB2*	Ventricular fibrillation without structural disease	Negative
Catecholaminergic polymorphic ventricular tachycardia	*RYR2, CASQ2, CALM1-3*	Stress-induced ventricular fibrillation/torsades de pointes	Negative
Short QT syndrome	*KCNH2, KCNQ1, KCNJ2*	Ventricular fibrillation without structural disease	Negative
Early repolarization syndrome	*CACNA1C, CACNB2, KCNJ8*	Idiopathic ventricular fibrillation pattern	Negative

### The Paradigm Shift: ILR as the Definitive Phenotype Arbiter

Although genetic testing excluded known causes, prolonged rhythm monitoring proved essential in phenotypic clarification. ILR interrogation documented that the captured rhythm was not TdP,^8^ but rather the abrupt onset of primary ventricular fibrillation (Figure 2). This distinction is critically important. TdP is typically pause dependent or bradycardia dependent,^9^ whereas primary ventricular fibrillation often arises abruptly without a clear initiating sequence. This ILR documentation supported a working diagnosis most consistent with IVF: a heart that can spontaneously initiate lethal arrhythmia without the specific electrophysiological cascade of other channelopathies based on currently available diagnostic modalities. This case argues for the mandatory use of long-term cardiac monitoring in all unexplained survivors of sudden cardiac death, not only for diagnosis of arrhythmic mechanism, but also for defining the dominant electrophysiological mechanism.

### Expanding the Phenotype: Seizure-Like Presentations and Purkinje-Mediated Triggers

In the broader literature, IVF has a heterogeneous clinical presentation, ranging from presyncope and syncope to seizure-like activity. Although convulsive syncope during ventricular fibrillation arrest is common, true seizure presentation as the initial manifestation is distinctly uncommon among published IVF cases.^10^ The initial misclassification of our patient’s condition as a neurologic event underscores the need to consider arrhythmic etiologies in atypical seizure presentations, particularly in young patients without a prior neurologic diagnosis.

Mechanistically IVF is triggered by short-coupled premature ventricular contractions (PVCs), often arising from the Purkinje network.^11^ This pattern was first described by Leenhardt et al,^12^ who identified the short-coupled variant of TdP, characterized by narrow PVCs with coupling intervals typically <400 ms.^13^ Similar electrophysiological behavior was noted in our case, reinforcing the mechanistic link to Purkinje-mediated triggers.

Haïssaguerre et al^14^ later demonstrated that Purkinje-triggered PVCs represent the dominant mechanism in IVF and showed that catheter ablation targeting these foci can reduce ventricular fibrillation recurrence by up to 89% on long-term follow-up. Notably, these studies also described microstructural myocardial abnormalities that may persist despite normal cardiac magnetic resonance results, highlighting that conventional imaging lacks the resolution to detect subtle arrhythmogenic substrates. In our patient, an electrophysiological study was performed with intent to map and ablate triggering PVCs; however, a low and inconsistent PVC burden during the procedure precluded reliable activation mapping. Although short-coupled PVCs were intermittently observed, no stable or reproducible focus could be identified, and ablation was not successful. This outcome underscores the heterogeneous and sometimes elusive substrate of IVF and highlights ongoing gaps in mapping precision and therapeutic strategies.

## Conclusions

This case illustrates that the path to diagnosing IVF is often nonlinear and requires vigilance beyond the initial presentation. The early findings suggestive of LQTS served as a diagnostic decoy, but a disciplined, exhaustive work-up and the critical evidence provided by long-term monitoring ultimately revealed the true nature of the disease. Continuous follow-up remains essential, as future discoveries may yet elucidate the specific molecular mechanisms underlying this idiopathic condition. Ongoing research into genetic underpinnings continues to refine our understanding and may lead to more personalized therapies in the future.

**Visual Summary:** Timeline of Case

Timeline	Events
Date of submission/day 1	A 23-year-old African-American woman with no significant prior medical history presented to the hospital after experiencing seizure-like activity. Continuous video electroencephalogram monitoring was initiated. Simultaneous telemetry and video electroencephalogram was performed and confirmed the presence of polymorphic ventricular tachycardia that was consistent with torsades de pointes. Subsequent electrocardiogram showed markedly prolonged QTc of 496 ms and long QT syndrome was diagnosed. Patient was started on nadolol at this time.
Day 30	The patient underwent coronary computed tomography angiography and cardiac magnetic resonance; results were entirely normal, showing normal biventricular size and function with no structural abnormalities or evidence of cardiomyopathy, scar, or inflammation.
Day 32	An implantable cardioverter-defibrillator was successfully implanted.
Day 120	The patient was readmitted to the hospital after experiencing another ventricular fibrillation episode status post implantable cardioverter-defibrillator shock. A full genetic work-up was done, which was negative. It was decided to conduct an electrophysiology study with possibility of targeted premature ventricular contraction ablation if there was a potential of frequent premature ventricular contractions. However, procedure was a failure due to lack of localizing premature ventricular contractions.
Day 150	The patient presented with a syncopal episode with a prodrome of dizziness and loss of consciousness. Implantable cardioverter-defibrillator device was interrogated, which revealed polymorphic ventricular tachycardia degenerating to ventricular fibrillation, and the patient was defibrillated after 22 seconds of the entire episode leading to restoration of sinus rhythm. At this time it was decided to initiate amiodarone.
Day 180	At 30-day follow-up, the patient had not experienced any ventricular arrhythmia events since initiating amiodarone.

## Funding Support and Author Disclosures

The authors have reported that they have no relationships relevant to the contents of this paper to disclose.
